# Genetic Evidence for Inhibition of Bacterial Division Protein FtsZ by Berberine

**DOI:** 10.1371/journal.pone.0013745

**Published:** 2010-10-29

**Authors:** Jaroslaw M. Boberek, Jem Stach, Liam Good

**Affiliations:** 1 Department of Pathology and Infectious Diseases, The Royal Veterinary College, University of London, London, United Kingdom; 2 School of Biology, University of Newcastle, Newcastle upon Tyne, United Kingdom; The Scripps Research Institute, United States of America

## Abstract

**Background:**

Berberine is a plant alkaloid that is widely used as an anti-infective in traditional medicine. *Escherichia coli* exposed to berberine form filaments, suggesting an antibacterial mechanism that involves inhibition of cell division. Berberine is a DNA ligand and may induce filamentation through induction of the SOS response. Also, there is biochemical evidence for berberine inhibition of the cell division protein FtsZ. Here we aimed to assess possible berberine mechanism(s) of action in growing bacteria using genetics tools.

**Methodology/Principal Findings:**

First, we tested whether berberine inhibits bacterial growth through DNA damage and induction of the SOS response. The SOS response induced by berberine was much lower compared to that induced by mitomycin C in an SOS response reporter strain. Also, cell filamentation was observed in an SOS-negative *E. coli* strain. To test whether berberine inhibits FtsZ, we assessed its effects on formation of the cell division Z-rings, and observed a dramatic reduction in Z-rings in the presence of berberine. We next used two different strategies for RNA silencing of *ftsZ* and both resulted in sensitisation of bacteria to berberine, visible as a drop in the Minimum Inhibitory Concentration (MIC). Furthermore, Fractional Inhibitory Concentration Indices (FICIs) showed a high level of synergy between *ftsZ* silencing and berberine treatment (FICI values of 0.23 and 0.25 for peptide nucleic acid- and expressed antisense RNA-based silencing of *ftsZ*, respectively). Finally, over-expression of *ftsZ* led to a mild rescue effect in berberine-treated cells.

**Conclusions:**

The results argue against DNA binding as the primary mechanism of action of berberine and support the hypothesis that its antibacterial properties are due to inhibition of the cell division protein FtsZ. In addition, the genetic approach used here provides a means to rapidly test the activity of other putative FtsZ inhibitors.

## Introduction

**Figure 1:**
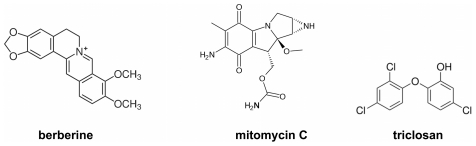
Chemical structures of antibacterial compounds used in this study.

Berberine (Figure 1) is an alkaloid produced by several plant species including barberry (*Berberis spp*.) and goldenseal (*Hydrastis spp.*), and is usually found in roots, stems and bark. Berberine has been shown to exhibit antibacterial activity against a variety of bacteria, including many pathogenic species and multidrug-resistant (MDR) strains of *M. tuberculosis* and MRSA. It is also active against some fungi and protozoans. Berberine is used in traditional Chinese, Native American and also in Western medicine, where it is recognised for its antimicrobial properties. In addition, recent studies have revealed tumoricidal and anti-inflammatory activities of the compound [1]–[9]. Berberine is a relatively weak antimicrobial, especially against Gram-negative bacteria. This is due to it being a substrate for the multi-drug resistance efflux pumps; the presence of MDR pump inhibitors remarkably increases the antibacterial effectiveness of the compound [10]. Despite much data for its antimicrobial activity, the mechanism of action of berberine in bacteria has remained unclear.

A number of studies demonstrate that berberine is a DNA ligand, able to bind both single- and double-stranded DNA *in vitro* [11]–[14]. Thus, berberine binding to DNA in bacteria could lead to DNA damage. If so, berberine would induce the bacterial SOS response - a multi-level post-replication DNA repair system. The SOS response involves over 20 genes and is regulated by LexA and RecA. Its purpose is, firstly, to detect DNA damage, activate repair mechanisms like NER (Nucleotide Excision Repair) and, if necessary, to prevent cell division through binding of FtsZ by SulA, resulting in cell elongation. This gives the cell time to repair DNA lesions by inducing a further repair mechanism involving the mutagenic DNA repair polymerase V.

An alternative possible mechanism of action for berberine was reported in a recent study [2] that provides *in vitro* evidence for inhibition of FtsZ, a protein that is key to bacterial cell division. FtsZ assembles into a contractile ring (called the Z-ring) at the midcell site of the future septum [15]. FtsZ is highly conserved among bacteria and is a prokaryotic homologue of eukaryotic tubulin [16], [17]. The Z-ring is formed of protofilaments of polymerised FtsZ subunits. FtsZ, like eukaryotic tubulin, is a GTPase and polymerises in a GTP-dependent manner. Z-ring formation is the earliest known step in bacterial cytokinesis. The mechanisms involved in Z-ring contraction are unclear, but FtsZ assembly into the Z-ring is essential to cell division and crucial to the recruitment of other division proteins including FtsA, ZipA, FtsK, FtsQ, AmiC and EnvC [18], [19]. Because of its vital role in cell division and its high level of conservation, FtsZ is a desirable target for antibacterial drug development. Several inhibitors have been found to date and these include both synthetic and natural products [20]–[25].

Previous studies on the mode of action of berberine were mainly biochemical and included NMR spectroscopy, FtsZ polymerisation and GTPase activity assays, fluorescence and electron microscopy. It was shown that berberine binds to FtsZ with high affinity, inhibits FtsZ assembly and its GTPase activity *in vitro*. Berberine causes cell elongation, which is an expected effect of cell division inhibition. However, cell elongation can be caused by other factors such as DNA damage and induction of the bacterial SOS response. Therefore, the antibacterial mode of action of berberine requires further elucidation.

There is evidence for both DNA binding and FtsZ inhibition as mechanisms that could explain the antibacterial activities of berberine. Both possibilities are supported by extensive *in vitro* data, but cellular and genetic evidence is lacking. Here we attempted to test both of these possible mechanisms in live, growing cells through functional assays. To test whether berberine binds and damages DNA, an *E. coli* SOS response reporter strain was used to report damage caused by berberine in growing cells. Also, an SOS response-negative strain was used to establish whether the cell elongation effect of berberine treatment is dependent on a functional SOS response system. To test whether berberine inhibits FtsZ, we tested the effects of berberine on FtsZ localisation and Z-ring assembly *in vivo*. Also, we used two types of RNA silencing to reduce *ftsZ* expression. RNA silencing is a powerful research tool that enables conditional and titratable expression reduction of specific genes and thus aids functional analyses. Here, we applied selective RNA silencers, based both on expressed antisense RNA and peptide nucleic acids (PNA), designed to target *ftsZ* mRNA [26] and also tested the effects of over-expression of *ftsZ* on susceptibility to berberine. The findings are consistent with berberine acting as an FtsZ inhibitor.

## Materials and Methods

### Bacteria, growth conditions and RNA silencing

*E. coli* wild-type strain K-12 in Mueller Hinton Broth (MHB, Fluka, Germany) was used for PNA (Panagene, Korea) studies. Overnight *E. coli* cultures were standardised by OD_550_ readings to approximately 10^5^ cfu/ml and then 20 µl was used as inoculum in each well of a 96-well plate (Costar, UK). PNA concentrations (0–2 µM) were optimised for a titration in growth inhibition. Overnight cultures of derivatives of TOP10 *E. coli* (Invitrogen, UK) carrying plasmids expressing antisense sequences (Table 1) were standardised as above. Antisense expression was induced by IPTG (0, 20, 40 or 70 µM) added to MHB supplemented with chloramphenicol (30 µg/ml). For antisense PNA treatment and IPTG induction, PNA (Table 2) or IPTG in aqueous solution was deposited into wells of a 96-well plate before the addition of 20 µl *E. coli* in liquid culture to a final volume of 200 µl (for a final 10^4^ cfu/ml). Bacterial cultures were grown in a Bio-Tek PowerWave X340I spectrophotometer at 37°C with agitation every 5 min. Growth was monitored by OD_550_ readings every 5 min. For *ftsZ* over-expression experiments, *E. coli* strain DH5α carrying plasmid pBAD-ftsZ (Table 1) was grown as described above with a series of L-arabinose concentrations. Each condition was performed in triplicate.

**Table 1:** Plasmids used in this study.

Plasmid	Relevant features	Purpose	Antisense target location* and length	Reference/source
pHNZ	pHN678 derivative, IPTG-inducible promoter (P_trc_), Cam^R^, *ftsZ* antisense insert	Inducible expression of *ftsZ* antisense	−53 to +76 of *ftsZ* (129 nt.)	[26]
pHN682	pHN678 derivative, IPTG-inducible promoter (P_trc_), Cam^R^, *fabI* antisense insert	Inducible expression of *fabI* antisense	−74 to +86 of *fabI* (160 nt.)	[51]
pLAU80	L-arabinose-inducible promoter P_BAD_, *ftsZ-yfp* fusion, Amp^R^	Inducible expression of *ftsZ-yfp* fusion	n/a	[49]
pBAD-ftsZ	L-arabinose-inducible promoter (P_BAD_), *ftsZ* ORF, Amp^R^	Inducible over-expression of *ftsZ*	n/a	[26]

*antisense target locations are indicated relative to the start codon.

**Table 2:** Structure of PNA used in this study.

PNA	Sequence	Target	Target location* and length	Reference/source
Ec326	(KFF)3K-eg1-tcaaacatag	*ftsZ*	−2 to +8 (10 nt.)	[26]
Ec107	(KFF)3K-eg1-cccatagctt	*fabI*	−5 to +5 (10 nt.)	[52]

*antisense target locations are indicated relative to the start codon.

### Minimum Inhibitory Concentration (MIC) determination

Berberine hemisulphate (Alexis Biochemicals, CA), triclosan (Ciba AG, Switzerland) and mitomycin C (Roche, UK) were added in a series of dilutions to growth medium containing approximately 10^4^ cfu/ml of bacteria. MIC was scored as the lowest concentration of an antimicrobial compound at which no growth of the tested *E. coli* strain was observed after 18 hours of incubation in a 96-well plate prepared as described above.

### *E. coli* SOS response studies

*E. coli* strain SS996 (Table 3) was grown for 18 hours in a 96-well plate in a Bio-Tek PowerWave X340I spectrophotometer as described above, and levels of GFP fluorescence were measured in a Spectramax M2 (Molecular Devices, CA) spectrophotometer using 488 nm (excitation) and 520 nm (emission) filters.

**Table 3:** *Escherichia coli* strains used in this study.

Strain	Relevant genotype	Relevant features	Purpose	Berberine MIC (mM)	Reference/source
K-12 (MG-1655)	n/a	wild-type strain	PNA silencing studies	1.5	Coli Genetic Stock Center
KG22	n/a	n/a	expression of *ftsZ-yfp* fusion	4	[49]
SS996	*Δattλ*::*sulApΩgfp-mut2*, *sulB*103	*sulA* promoter-*gfp* fusion, *ftsZ* allele insensitive to SulA	SOS response studies	4	[29]
KP7600	W3110 derivative miniTn10, Kan^R^	wild-type strain	reference strain for JD26285	4.5	[50]
JD26285	*ΔsulA*773::*kan*, KP7600 derivative, Kan^R^	negative for SOS-mediated cell division inhibition (*sulA* ^−^)	expression of antisense RNA; cell morphology studies	4.5	[50]
DH5α	n/a	n/a	cloning and expression of *ftsZ*	2.5	Invitrogen

### Fluorescence microscopy

Overnight cultures of *E. coli* KP7600 (wild-type) and JD26285 (*sulA*::Tn5 SOS^−^ mutant, see Table 3) were grown in MHB, refreshed 1∶50 with fresh MHB medium and then either untreated or treated with different concentrations of berberine hemisulphate (1.5, 2.5 and 3 mM) for 2 hours at 37°C and 200 rpm. The cells were viewed by fluorescence microscopy at 630× total magnification on a DM 4000B fluorescence microscope (Leica, Germany).

For Z-ring formation studies, *E. coli* strain KG22-pLAU80 (a gift from Professor Kenn Gerdes and Ms Elisa Galli, Newcastle University) containing plasmid pLAU80 (Table 1) carrying a *ftsZ-yfp* fusion was used. An overnight culture was refreshed as above and treated with 3 mM berberine for one hour. 0.2% L-arabinose was added in order to induce production of FtsZ-YFP. After one hour, induction was stopped by addition of 0.2% glucose and the cells were allowed to grow for another 30 minutes. The culture was then harvested by centrifugation; washed three times in an equal volume of 1 × PBS in order to remove the residues of growth medium and berberine, and re-suspended in 1 ml of 1 × PBS. DAPI (Sigma, UK) was added to a final concentration of 1 µM, cells were incubated at room temperature for 10 mins, washed three times with 1 × PBS and viewed under a fluorescent microscope as above. Images were captured and processed using Leica IM 500 and Adobe Photoshop Elements software.

### Fractional Inhibitory Concentration Indices (FICIs) calculations for synergy studies

Fractional Inhibitory Concentrations (FICs) for berberine and the applied gene silencers were calculated as follows:
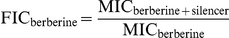


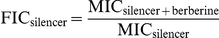



FICIs were determined by adding the FIC values for berberine and each silencer:
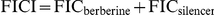



The FICIs represent the level of paired interaction, where ‘synergy’ is defined by values ≤0.5 and ‘no interaction’ by values >0.5–4.0 [27]. The combinations of berberine and silencer were tested in triplicate.

### Statistical analysis

Statistical analyses of R^2^ for correlation, mean ± SD calculations and ANOVA were carried out using MS Excel 2007.

## Results

### Berberine does not induce a significant SOS response in *E. coli*

**Figure 2:**
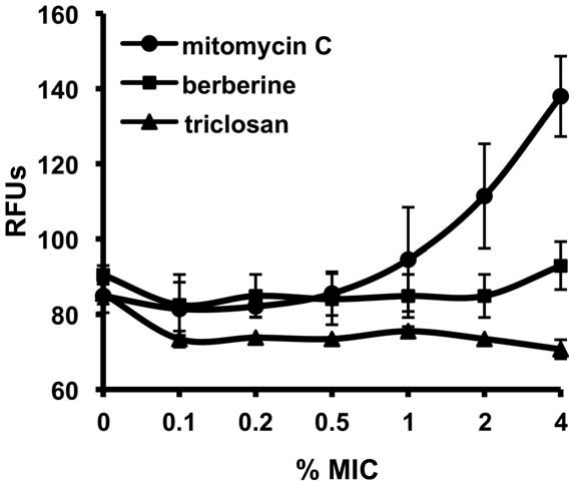
Bacterial SOS response in the presence of berberine. The doses of each compound were chosen as indicated percentage of the MIC (4 mM, 15 nM and 400 nM for berberine, mitomycin C and triclosan, respectively). Mitomycin C and triclosan were included as positive and negative controls for SOS induction. The Relative Fluorescence Units (RFUs) indicate the level of SOS reporter expression in the SOS-negative *E. coli* strain SS996.

Berberine has been reported to be a DNA ligand and some of the effects it has on bacterial cells, particularly cell elongation could be explained by DNA binding/damage and subsequent induction of the bacterial SOS response. Hence, to test the possibility that the mode of action of berberine is a result of its ability to bind DNA, *E.* coli SS996, an SOS response indicator strain, was used. This strain contains a fusion of the promoter of the SOS response division inhibitor gene *sulA* with *gfp* and also an allele of FtsZ that is insensitive to SulA (SulB103) [28]. The strain is negative for SOS-induced cell division inhibition, but the presence of the *sulAp-gfp* fusion allows a measurement of the level of fluorescence, and thereby SOS response induction in the cell caused by a test compound [29]. *E. coli* SS996 was grown with the addition of different amounts of berberine. For comparison, equal amounts of mitomycin C and triclosan (as a respective percentage of the MIC of each compound which did not cause a significant growth inhibition) were used. Mitomycin C, a potent DNA crosslinker used as an anti-tumour agent, was applied as a positive control. Triclosan, a specific inhibitor of the enoyl-acyl carrier protein reductase (FabI) involved in fatty acid biosynthesis, was used as a negative control. Relative fluorescence of *E. coli* SS996 treated with the three compounds is presented in Figure 2. Both triclosan and berberine induced virtually no SOS response at doses of up to 4% of their MIC. In contrast, mitomycin C caused a significant level of fluorescence in this dose range, indicating strong induction of the SOS response. The results suggest that berberine induction of the SOS response in bacteria is very low relative to mitomycin C.

### Berberine treatment results in cell elongation in an SOS response-negative strain

**Figure 3:**
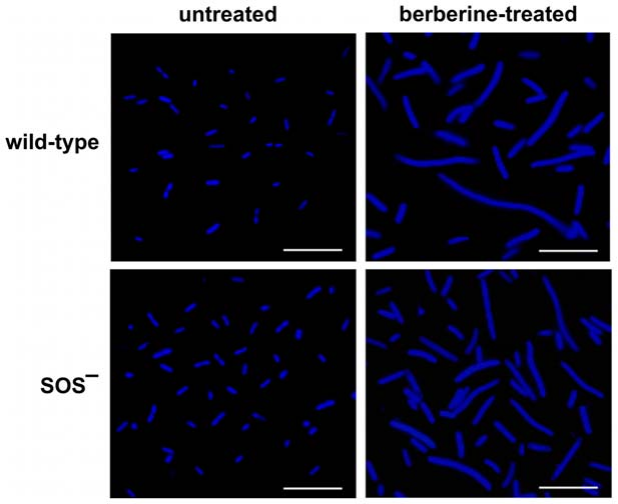
Effect of berberine on *E. coli* morphology. Bacteria (wild-type and the SOS-negative strain) were either untreated or treated with 1.5 mM berberine. Scale bars are 10 µm.

If berberine causes cell elongation through inhibition of the bacterial division protein FtsZ and not induction of the SOS response, then berberine should also cause elongation in an SOS response-negative strain. To test this possibility, *E. coli* strain JD26285 (SulA^−^, SOS-negative) and KP7600 (parent strain, wild-type, Table 3) were cultured either with or without sub-inhibitory concentrations of berberine and examined using fluorescence microscopy. Untreated cells exhibited normal morphology, whereas berberine-treated cells of both strains were significantly elongated (Figure 3). Thus, berberine also causes cell elongation in a strain lacking the SOS response. Together these results argue strongly against the hypothesis that the antimicrobial effect of berberine is due to DNA binding activity.

### Berberine inhibits Z-ring formation *in vivo*

**Figure 4:**
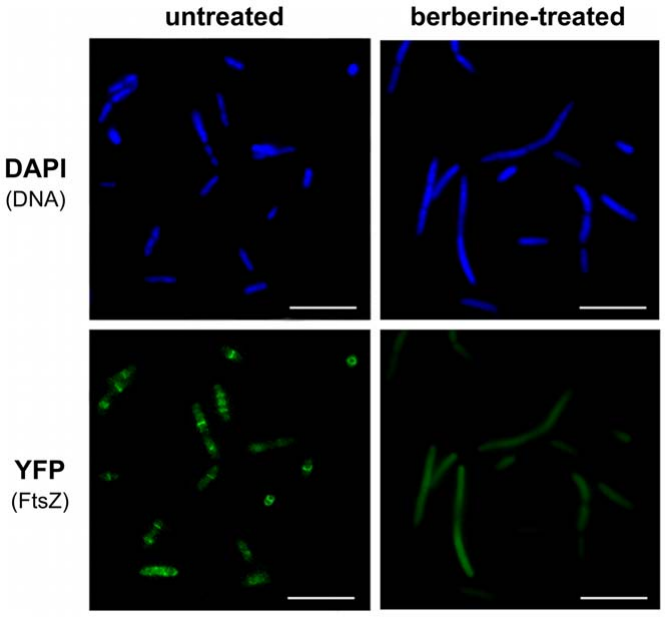
Z-ring formation and cell morphology in berberine-treated *E. coli*. Representative cells untreated and treated with 3 mM berberine; FtsZ was tagged with YFP and bacterial DNA was visualised through staining with DAPI. Scale bars are 10 µm.

To study the effect of berberine on Z-ring formation, we applied an *E. coli* strain KG22 carrying pLAU80 (Table 1), which contains an *ftsZ-yfp* fusion. In the absence of berberine, clear Z-rings were visible in many of the observed cells (Figure 4), and some cells exhibited multiple Z-rings and an elongated structure, presumably due to over-abundance or dysfunction of the FtsZ-GFP fusion [30], [31]. Most cells displayed one or more Z-rings. Addition of 3 mM berberine heavily perturbed the formation of the Z-rings. The fluorescence was evenly distributed in the cytoplasm of the berberine-treated bacteria, and very few cells displayed the Z-rings. These findings also support the hypothesis that FtsZ inhibition is the main mode of action of berberine in bacteria.

### Sensitisation of *E. coli* to berberine through *ftsZ* silencing

**Figure 5:**
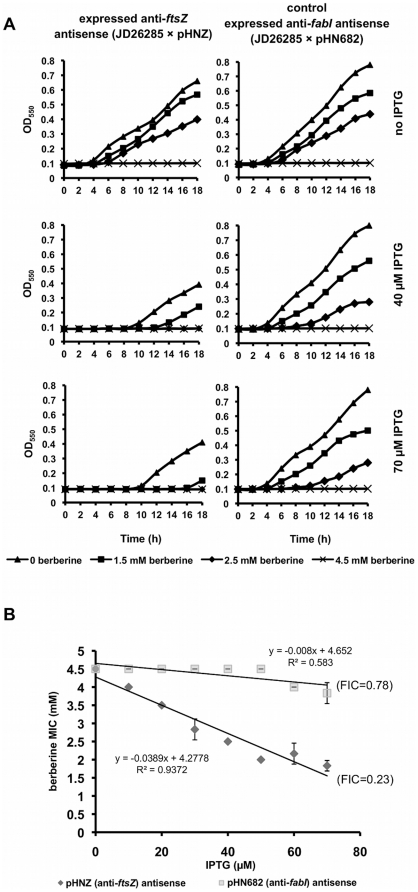
Effect of expressed antisense RNA-mediated *ftsZ* silencing on *E. coli* growth and susceptibility to berberine. (A) Growth curves for the SOS-negative strains carrying the control and anti-*ftsZ* plasmids. Antisense expression was induced by a range of IPTG concentrations (0–70 µM) and cells were treated with the indicated concentrations of berberine. (B) Regression analysis of IPTG concentration and the MIC of berberine in both strains.

To examine the mode of action of berberine using gene expression titration experiments, we applied two different, established methods of RNA silencing: expressed antisense RNA and antisense PNA. The specificity of these methods has been examined in several studies and validated silencers have been reported for the *E. coli ftsZ* gene [26]. *E. coli* growth was monitored both in the presence and absence of berberine, and with or without *ftsZ* silencing using PNA and antisense RNA. This strategy was based on the principle that lowering (but not eliminating) the expression of a target gene using antisense technology would cause the bacteria to be more susceptible to an antimicrobial compound targeting the product of that gene. In the case of expressed antisense RNA, *E. coli* SOS-negative strain JD26285 carrying either plasmid pHNZ (anti-*ftsZ*), or pHN682 (anti-*fabI* antisense, control) were grown in the presence of a range of berberine and IPTG (inducer for the antisense RNA expression) concentrations. The growth curves and MICs for different levels of anti-*ftsZ* RNA expression are presented in Figure 5. Addition of IPTG had a clear inhibitory effect on the growth of cells due to silencing of a growth-essential gene. Silencing of *ftsZ* prolonged the lag phase and lowered the final OD. Moreover, silencing of *ftsZ* caused the bacteria to be more sensitive to berberine. Most importantly, upon induction of antisense anti-*ftsZ* RNA expression, bacterial growth was completely inhibited at a lower concentration of berberine. Silencing of *ftsZ* expression resulted in a reduction of the MIC of berberine from 4.5 mM without IPTG to 2 mM for 70 µM IPTG, whereas the control strain was not affected by IPTG (MICs were the same within the applied range of IPTG concentrations). In addition, the FIC indices revealed synergy between anti-*ftsZ* RNA expression and berberine treatment (FICI = 0.23, Fig. 5) and suggest no interaction between berberine addition and silencing of the control gene *fabI* (FICI = 0.78).

**Figure 6:**
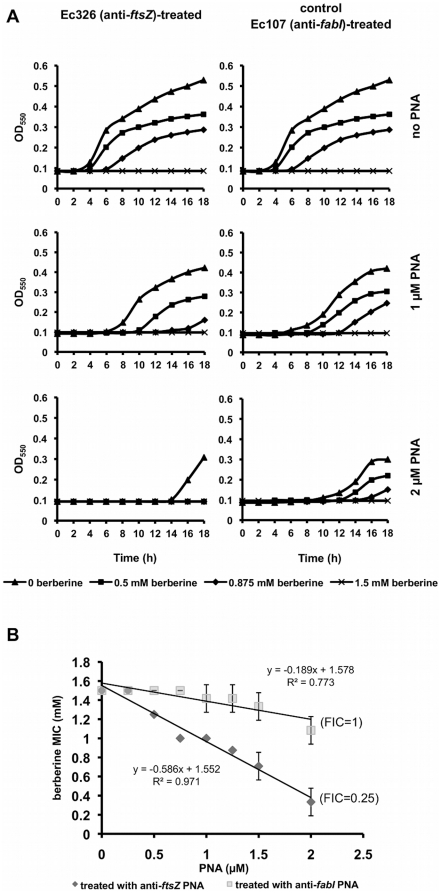
Effect of PNA-mediated *ftsZ* silencing on cell growth and susceptibility to berberine in *E. coli* K-12 strain. (A) Typical growth curves; cells were grown in the presence one of two different PNAs: Ec326 (anti-*ftsZ*) or Ec107 (anti-*fabI*, negative control) in a range of concentrations (0–2 µM) and treated with the indicated concentrations of berberine. (B) Regression analysis of PNA concentration and the MIC of berberine.

Addition of anti-*ftsZ* PNA had an effect on bacterial growth that was similar to that observed with expression of anti-*ftsZ* RNA. In this case wild-type K-12 *E. coli* strain and PNA targeting *ftsZ* (Ec326) and *fabI* (Ec107, control) were utilised. Again, bacteria were sensitised to berberine when the *ftsZ* gene was silenced by the PNA; the MIC of berberine was reduced from 1.75 mM (no PNA added) to 0.25 mM (2 µM Ec326 added). As a control, silencing of *fabI* had no effect on cell susceptibility to berberine. Also, FIC indices suggest synergy between silencing of *ftsZ* and berberine treatment (FICI = 0.25) compared with FICI = 1 for silencing of *fabI*. Growth curves and linear regression analysis of MIC is shown in Figure 6.

The results from both silencing approaches support the hypothesis that berberine targets the bacterial division protein FtsZ. For comparison, the MICs of berberine for each of the *E. coli* strains used in this study are presented in Table 3.

### Antagonism between *ftsZ* over-expression and berberine effect on bacterial growth

**Figure 7:**
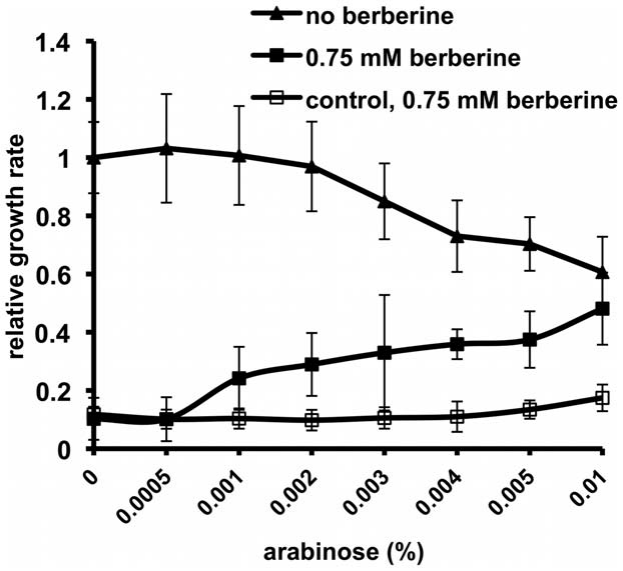
Over-expression of *ftsZ* and its effect on *E. coli* growth in the presence and absence of berberine. Bacteria (*E. coli* DH5α carrying plasmid pBAD-ftsZ) were induced using a concentration range of L-arabinose (0–0.01%) to allow over-expression of the FtsZ protein and grown in the absence or presence of berberine. The growth rate in the absence of L-arabinose or berberine was set as 1. As a control, *E. coli* DH5α without the plasmid was treated with berberine in the same range of L-arabinose concentrations.

To establish whether over-expression of *ftsZ* increases bacterial resistance to berberine, we used *E. coli* DH5α carrying plasmid pBAD-ftsZ, and DH5α without a plasmid was used to prove a control having basal *ftsZ* expression. Relative growth rates were calculated as ΔOD/Δt, and cultures were scored after Δt = 10 hours, as further *ftsZ* over-expression was growth-inhibitory (data not shown). In the case of the strain over-expressing *ftsZ*, without the addition of berberine, an increase in L-arabinose concentration caused a growth rate decrease. This was because *ftsZ* over-expression is toxic to the cell, presumably due to its crucial and multi-faceted role in cell division [32], [26], [33], [34]. In the presence of arabinose, the addition of berberine increased bacterial growth (Figure 7); the cultures exhibited a moderate increase in the relative growth rate with increasing amounts of L-arabinose. In the case of the control strain, addition of L-arabinose did not rescue bacterial growth from inhibition by berberine. These results indicate that *ftsZ* over-expression antagonised the toxic effect of berberine, further suggesting that berberine targets FtsZ.

## Discussion

The plant-derived isoquinoline alkaloid berberine has a long history of use as an antimicrobial agent. Also, it has been shown to suppress the growth of a variety of tumour cells, and is effective in certain types of diabetes or cardiovascular conditions [6], [35]–[44]. Understanding the mechanism(s) of action of berberine is of significant interest and requires further studies. Berberine is a known DNA and RNA binder [45], [11], [46], [47], and recently it was reported to target the bacterial division protein FtsZ [2]. The evidence supporting these findings was obtained mainly through biochemical and biophysical studies. Here, we aimed to test these two hypotheses for the mode of action of berberine in bacteria using a molecular genetics approach. Firstly, we investigated the possibility that the growth inhibitory properties of berberine are due to its ability to bind to DNA and, therefore, induced bacterial SOS response. Within the studied range of concentrations, berberine did not cause a significant level of SOS response in the indicator *E. coli* strain SS996 relative to the positive control. This result is consistent with an earlier study of berberine using the SOS chromotest, which showed that the compound is not a potent genotoxic or mutagenic agent [48]. Furthermore, berberine caused cell elongation in an SOS-negative strain, as it did in the wild-type strain. Fluorescence microscopy showed berberine to cause disruptions of Z-ring formation *in vivo*. These findings suggested that FtsZ is the main target for berberine in bacteria. To further test this hypothesis, we studied the relationship between the cellular abundance of the putative target and the effect of berberine on bacterial growth; *ftsZ* expression was titrated using antisense and over-expression tools. Lowering the expression of *ftsZ* resulted in increased susceptibility of the bacteria to berberine and also in a decrease in the MIC. In the control assay, upon silencing of another essential gene, *fabI,* sensitisation to berberine was not observed. Furthermore, over-expression of *ftsZ*, despite high level of toxicity, rescued the growth of berberine-treated cells.

In conclusion, the data argue against DNA binding/damage as the primary mechanism of action of berberine, and argue strongly for inhibition of FtsZ as the primary target for berberine in bacteria. This does not exclude the possibility that berberine can act in part through DNA or RNA binding or by inhibition of other cell division proteins such as FtsA or ZipA. Several possible mechanisms of action of berberine in mammalian cells have been proposed. Nevertheless, the evidence that berberine inhibits FtsZ clarifies how this compound exerts an antibacterial effect. Berberine is used in traditional Chinese, Native American and Western medicine, and it may be possible to develop berberine analogues with improved potency. Finally, the RNA silencing strategies applied in this study provide a means to rapid identification of other FtsZ inhibitors.

## References

[1] Amin AH, Subbaiah TV, Abbasi KM (1969). Berberine sulfate: antimicrobial activity, bioassay, and mode of action.. Can J Microbiol.

[2] Domadia PN, Bhunia A, Sivaraman J, Swarup S, Dasgupta D (2008). Berberine targets assembly of Escherichia coli cell division protein FtsZ.. Biochemistry.

[3] Gentry EJ, Jampani HB, Keshavarz-Shokri A, Morton MD, Velde DV (1998). Antitubercular natural products: berberine from the roots of commercial Hydrastis canadensis powder. Isolation of inactive 8-oxotetrahydrothalifendine, canadine, beta-hydrastine, and two new quinic acid esters, hycandinic acid esters-1 and -2.. J Nat Prod.

[4] Hwang BY, Roberts SK, Chadwick LR, Wu CD, Kinghorn AD (2003). Antimicrobial constituents from goldenseal (the Rhizomes of Hydrastis canadensis) against selected oral pathogens.. Planta Med.

[5] Mahady GB, Pendland SL, Stoia A, Chadwick LR (2003). In vitro susceptibility of Helicobacter pylori to isoquinoline alkaloids from Sanguinaria canadensis and Hydrastis canadensis.. Phytother Res.

[6] Mantena SK, Sharma SD, Katiyar SK (2006). Berberine, a natural product, induces G1-phase cell cycle arrest and caspase-3-dependent apoptosis in human prostate carcinoma cells.. Mol Cancer Ther.

[7] Serafim TL, Oliveira PJ, Sardao VA, Perkins E, Parke D (2008). Different concentrations of berberine result in distinct cellular localization patterns and cell cycle effects in a melanoma cell line.. Cancer Chemother Pharmacol.

[8] Villinski J, Dumas E, Chai H, Pezzuto J, Angerhofer C (2003). Antibacterial Activity and Alkaloid Content of Berberis thunbergii, Berberis vulgaris and Hydrastis canadensis.. Pharmaceutical Biology.

[9] Yu H, Kim K, Cha J, Kim H, Lee Y (2005). Antimicrobial activity of berberine alone and in combination with ampicillin or oxacillin against methicillin-resistant Staphylococcus aureus.. J Med Food.

[10] Tegos G, Stermitz FR, Lomovskaya O, Lewis K (2002). Multidrug pump inhibitors uncover remarkable activity of plant antimicrobials.. Antimicrob Agents Chemother.

[11] Das S, Kumar GS, Ray A, Maiti M (2003). Spectroscopic and thermodynamic studies on the binding of sanguinarine and berberine to triple and double helical DNA and RNA structures.. J Biomol Struct Dyn.

[12] Li W, Lu H, Xu C, Zhang J, Lu Z (1998). Spectroscopic and Binding Properties of Berberine to DNA - and Its Application to DNA - Detection.. Spectroscopy Letters: An International Journal for Rapid Communication.

[13] Bhadra K, Maiti M, Kumar GS (2008). Berberine-DNA complexation: new insights into the cooperative binding and energetic aspects.. Biochim Biophys Acta.

[14] Yadav RC, Kumar GS, Bhadra K, Giri P, Sinha R (2005). Berberine, a strong polyriboadenylic acid binding plant alkaloid: spectroscopic, viscometric, and thermodynamic study.. Bioorg Med Chem.

[15] Bi EF, Lutkenhaus J (1991). FtsZ ring structure associated with division in Escherichia coli.. Nature.

[16] Nogales E, Downing KH, Amos LA, Löwe J (1998). Tubulin and FtsZ form a distinct family of GTPases.. Nat Struct Biol.

[17] Erickson HP (1995). FtsZ, a prokaryotic homolog of tubulin?. Cell.

[18] Margolin W (2005). FtsZ and the division of prokaryotic cells and organelles.. Nat Rev Mol Cell Biol.

[19] Weiss DS (2004). Bacterial cell division and the septal ring.. Mol Microbiol.

[20] Beuria TK, Santra MK, Panda D (2005). Sanguinarine blocks cytokinesis in bacteria by inhibiting FtsZ assembly and bundling.. Biochemistry.

[21] Domadia P, Swarup S, Bhunia A, Sivaraman J, Dasgupta D (2007). Inhibition of bacterial cell division protein FtsZ by cinnamaldehyde.. Biochem Pharmacol.

[22] Jaiswal R, Beuria TK, Mohan R, Mahajan SK, Panda D (2007). Totarol inhibits bacterial cytokinesis by perturbing the assembly dynamics of FtsZ.. Biochemistry.

[23] Lock RL, Harry EJ (2008). Cell-division inhibitors: new insights for future antibiotics.. Nat Rev Drug Discov.

[24] Margalit DN, Romberg L, Mets RB, Hebert AM, Mitchison TJ (2004). Targeting cell division: small-molecule inhibitors of FtsZ GTPase perturb cytokinetic ring assembly and induce bacterial lethality.. Proc Natl Acad Sci U S A.

[25] Wang J, Galgoci A, Kodali S, Herath KB, Jayasuriya H (2003). Discovery of a small molecule that inhibits cell division by blocking FtsZ, a novel therapeutic target of antibiotics.. J Biol Chem.

[26] Goh S, Boberek JM, Nakashima N, Stach J, Good L (2009). Concurrent growth rate and transcript analyses reveal essential gene stringency in Escherichia coli.. PLoS ONE.

[27] Odds FC (2003). Synergy, antagonism, and what the chequerboard puts between them.. J Antimicrob Chemother.

[28] Bi E, Lutkenhaus J (1993). Cell division inhibitors SulA and MinCD prevent formation of the FtsZ ring.. J Bacteriol.

[29] McCool JD, Long E, Petrosino JF, Sandler HA, Rosenberg SM (2004). Measurement of SOS expression in individual Escherichia coli K-12 cells using fluorescence microscopy.. Mol Microbiol.

[30] Pichoff S, Lutkenhaus J (2001). Escherichia coli division inhibitor MinCD blocks septation by preventing Z-ring formation.. J Bacteriol.

[31] Salimnia H, Radia A, Bernatchez S, Beveridge TJ, Dillon JR (2000). Characterization of the ftsZ cell division gene of Neisseria gonorrhoeae: expression in Escherichia coli and N. gonorrhoeae.. Arch Microbiol.

[32] Dziadek J, Madiraju MVVS, Rutherford SA, Atkinson MAL, Rajagopalan M (2002). Physiological consequences associated with overproduction of Mycobacterium tuberculosis FtsZ in mycobacterial hosts.. Microbiology (Reading, Engl.).

[33] Haney SA, Glasfeld E, Hale C, Keeney D, He Z (2001). Genetic analysis of the Escherichia coli FtsZ.ZipA interaction in the yeast two-hybrid system. Characterization of FtsZ residues essential for the interactions with ZipA and with FtsA.. J Biol Chem.

[34] Honrubia-Marcos MP, Ramos A, Gil JA (2005). Overexpression of the ftsZ gene from Corynebacterium glutamicum (Brevibacterium lactofermentum) in Escherichia coli.. Can J Microbiol.

[35] Choi MS, Yuk DY, Oh JH, Jung HY, Han SB (2008). Berberine inhibits human neuroblastoma cell growth through induction of p53-dependent apoptosis.. Anticancer Res.

[36] Ho Y, Lu C, Yang J, Chiang J, Li T (2009). Berberine induced apoptosis via promoting the expression of caspase-8, -9 and -3, apoptosis-inducing factor and endonuclease G in SCC-4 human tongue squamous carcinoma cancer cells.. Anticancer Res.

[37] Hur J, Hyun M, Lim S, Lee W, Kim D (2009). The combination of berberine and irradiation enhances anti-cancer effects via activation of p38 MAPK pathway and ROS generation in human hepatoma cells.. J Cell Biochem.

[38] Jeong HW, Hsu KC, Lee J, Ham M, Huh JY (2009). Berberine suppresses proinflammatory responses through AMPK activation in macrophages.. Am J Physiol Endocrinol Metab.

[39] Katiyar SK, Meeran SM, Katiyar N, Akhtar S (2009). p53 Cooperates berberine-induced growth inhibition and apoptosis of non-small cell human lung cancer cells in vitro and tumor xenograft growth in vivo.. Mol Carcinog.

[40] Kim JB, Yu J, Ko E, Lee K, Song AK (2009). http://www.ncbi.nlm.nih.gov/pubmed/19800775.

[41] Turner N, Li J, Gosby A, To SWC, Cheng Z (2008). Berberine and its more biologically available derivative, dihydroberberine, inhibit mitochondrial respiratory complex I: a mechanism for the action of berberine to activate AMP-activated protein kinase and improve insulin action.. Diabetes.

[42] Yin J, Gao Z, Liu D, Liu Z, Ye J (2008). Berberine improves glucose metabolism through induction of glycolysis.. Am J Physiol Endocrinol Metab.

[43] Yin J, Xing H, Ye J (2008). Efficacy of berberine in patients with type 2 diabetes mellitus.. Metab Clin Exp.

[44] Zhang H, Wei J, Xue R, Wu J, Zhao W (2010). Berberine lowers blood glucose in type 2 diabetes mellitus patients through increasing insulin receptor expression.. Metabolism.

[45] Bhadra K, Maiti M, Kumar GS (2008). Berberine-DNA complexation: new insights into the cooperative binding and energetic aspects.. Biochim Biophys Acta.

[46] Giri P, Suresh Kumar G (2010). Molecular recognition of poly(A) targeting by protoberberine alkaloids: in vitro biophysical studies and biological perspectives.. Mol Biosyst.

[47] Islam MM, Suresh Kumar G (2009). RNA-binding potential of protoberberine alkaloids: spectroscopic and calorimetric studies on the binding of berberine, palmatine, and coralyne to protonated RNA structures.. DNA Cell Biol.

[48] Pasqual MS, Lauer CP, Moyna P, Henriques JA (1993). Genotoxicity of the isoquinoline alkaloid berberine in prokaryotic and eukaryotic organisms.. Mutat Res.

[49] Lau IF, Filipe SR, Søballe B, Økstad O, Barre F (2003). Spatial and temporal organization of replicating Escherichia coli chromosomes.. Molecular Microbiology.

[50] Kitagawa M, Ara T, Arifuzzaman M, Ioka-Nakamichi T, Inamoto E (2005). Complete set of ORF clones of Escherichia coli ASKA library (a complete set of E. coli K-12 ORF archive): unique resources for biological research.. DNA Res.

[51] Nakashima N, Tamura T, Good L (2006). Paired termini stabilize antisense RNAs and enhance conditional gene silencing in Escherichia coli.. Nucleic Acids Res.

[52] Dryselius R, Nekhotiaeva N, Good L (2005). Antimicrobial synergy between mRNA- and protein-level inhibitors.. J Antimicrob Chemother.

